# A Proposal for a Comprehensive Grading of Parkinson's Disease Severity Combining Motor and Non-Motor Assessments: Meeting an Unmet Need

**DOI:** 10.1371/journal.pone.0057221

**Published:** 2013-02-27

**Authors:** Kallol Ray Chaudhuri, Jose Manuel Rojo, Anthony H. V. Schapira, David J. Brooks, Fabrizio Stocchi, Per Odin, Angelo Antonini, Richard J. Brown, Pablo Martinez-Martin

**Affiliations:** 1 National Parkinson Foundation Centre of Excellence, Kings College Hospital and Kings College, and University Hospital Lewisham, London, United Kingdom; 2 Department of Statistics, Centre of Human and Social Sciences, Spanish Council for Scientific Research, Madrid, Spain; 3 Institute of Neurology, University College London, London, United Kingdom; 4 Department of Medicine, Imperial College London, London, United Kingdom; 5 Department of Neurology, IRCCS San Raffaele, Rome, Italy; 6 Department of Neurology, Lund University Hospital, Lund, Sweden; 7 Department for Parkinson's Disease, IRCCS San Camillo, Venice, Italy; 8 Department of Psychology, Institute of Psychiatry, Kings College London, London, United Kingdom; 9 Alzheimer Disease Research Unit and CIBERNED, CIEN Foundation, Carlos III Institute of Health, Alzheimer Centre Reina Sofia Foundation, Madrid, Spain; Centre Hospitalier Universitaire Vaudois (CHUV), Switzerland

## Abstract

**Background:**

Non-motor symptoms are present in Parkinson's disease (PD) and a key determinant of quality of life. The Non-motor Symptoms Scale (NMSS) is a validated scale that allows quantifying frequency and severity (burden) of NMS. We report a proposal for using NMSS scores to determine levels of NMS burden (NMSB) and to complete PD patient classification.

**Methods:**

This was an observational, cross-sectional international study of 935 consecutive patients. Using a distribution of NMSS scores by quartiles, a classification based on levels from 0 (no NMSB at all) to 4 (very severe NMSB) was obtained and its relation with Hoehn and Yahr (HY) staging, motor and health-related quality of life scales was analyzed. Concordance between NMSB levels and grouping based on clinician's global impression of severity, using categorical regression, was determined. Disability and HRQoL predictors were identified by multiple regression models.

**Results:**

The distribution of motor and QoL scales scores by HY and NMSB levels was significantly discriminative. The difference in the classification of cases for both methods, HY and NMSB, was significant (gamma = 0.45; ASE = 0.032). Concordance between NMSB and global severity-based levels from categorical regression was 91.8%, (kappa_w_ = 0.97). NMS score was predictor of disability and QoL.

**Conclusions:**

Current clinical practice does not address a need for inclusion of non-motor scores in routine assessment of PD in spite of the overwhelming influence of NMS on disability and quality of life. Our data overcome the problems of “pure motor assessment” and we propose a combined approach with addition of NMSB levels to standard motor assessments.

## Introduction

Parkinson's disease (PD) is a progressive, complex disorder characterised by motor symptoms, such as bradykinesia, rest tremor, and rigidity but also a wide range of non-motor symptoms (NMS) that contribute to significant morbidity and disability. NMS such as sleep dysfunction, dementia and depression are key determinants of patients' health-related quality of life (HRQoL) [1], [2]. Furthermore, while motor heterogeneity of PD is well established, clinical subtyping of Parkinson's based on non-motor symptoms has not been clearly established [3]–[6]. A pathological basis of non-motor endophenotypes has been suggested while subtyping of PD using latent class analysis indicate clusters with varying and sometimes dominant non-motor load [4], [5], [7]. Furthermore, a distinct phase (phase 2) associated with specific non-motor symptoms has been proposed in the natural history of PD [6], [8].)

Currently, PD severity is often rated using Hoehn and Yahr staging (HY) which purely reflects the motor severity of disease and compromise of balance/gait. The diagnosis of PD is made using the UK Parkinson's Disease Society Brain Bank clinical diagnostic criteria based exclusively on specific motor symptoms, mainly related to substantia nigra pathology [9], [10]. Interestingly, NMS such as sleep, autonomic and executive dysfunction, pain, and fatigue, may occur early or at a pre-motor phase and increase in prevalence and severity over time [11]–[15]. Importantly, it is not one NMS but a combination of several NMS that may serve in future to underpin refining diagnosis and management of PD [15].

In the clinic, current practice does not include obligatory assessment of non-motor scores using validated scales in spite of the overwhelming effect of NMS on quality of life. As a result, many NMS may remain undetected and lead to suboptimal care as reported from a recent European survey [16]. In this article, we provide the framework for improving our clinical assessment of PD in the clinic by incorporating a “snapshot” burden of NMS score to the existing motor assessments. Specific levels of burden of NMS (NMSB) are described based on an analysis of detailed NMS data available from an international database of consecutive PD cases. This pragmatic and score-based assessment paradigm can be easily adopted in the clinic taking into account both the severity of the motor and non-motor burden to improve the current system of classification.

## Methods

### Design

This was an observational, multi centre, cross-sectional, international study.

### Patients

Data from 951 consecutive PD patients diagnosed by a neurologist/geriatrician (movement disorders specialists) according to international recognized diagnostic criteria [10], [14] were included in the multipurpose database built and secured in the Alzheimer Centre Reina Sofia Foundation, Carlos III Institute of Health, Madrid.

Exclusion criteria were: Atypical and secondary parkinsonism (multiple system atrophy, progressive supranuclear palsy, etc); concomitant severe systemic disease (e.g., clinically severe organ failure such as cardiac failure, hepatic failure) or condition interfering with assessments required for study (e.g., blindness); inability to read, understand, or answer written questionnaires, or inability to provide informed consent. Overt dementia impeding evaluation, as per the clinical judgment, was a specific exclusion criterion.

#### Data

The data were collected from two independent series collected by the main authors (KRC and PMM) using a common protocol for clinical assessments and data capture. The bulk of the data used for the present study arose from a cross-sectional study designed to validate the NMS scale and data at baseline from a long-term international longitudinal study addressing the natural history of PD non-motor symptoms and has been published previously [2], [17].

#### Setting and locations

Departments of Neurology and Movement Disorder Units from centres in 15 countries of America, Asia, and Europe (ref. 17 and Annex S1).

#### Dates

The final database was built from data collected from 2007–2011.

### Ethical aspects

The non-motor scale validation studies received ethical approval from Carlos III Institute of Health, Madrid, Spain [2], [17] and research ethics committee at University Hospital Lewisham, London, UK [18]. The longitudinal NMS natural history study has been approved in all relevant institutions and is included in UK Department of Health portfolio of approved studies. All participant researchers obtained approval from their respective local EC/IRB and patients signed their informed consent before inclusion.

### Assessments

In addition to socio-demographic and historical data, the following instruments were applied:

The Scales for Outcomes in Parkinson's Disease-Motor (SCOPA-Motor), has 3 sections: A. Examination (10 items); B. Activities of daily living (ADL, 7 items); and C. Complications (4 items). Possible responses per item range from 0 (normal) to 3 (severe) with total score between 0 and 75 [19], [20].

The Non-Motor Symptoms Scale (NMSS) has 30 items, nine domains: cardiovascular (2 items), sleep/fatigue (4 items), mood/cognition (6 items), perceptual problems/hallucinations (3 items), attention/memory (3 items), gastrointestinal tract (3 items), urinary function (3 items), sexual function (2 items), and miscellaneous (4 items). Each item scores on a multiple of severity (from 0 to 3) and frequency scores (from 1 to 4) and the theoretical range of the NMSS total score is 0 to 360 [17], [18]. We refer to this score as “burden” (NMSB) since values integrate frequency and severity.

The original Hoehn and Yahr (HY) classification was used in this study [9].

The Clinical Impression of Severity Index (CISI-PD) addresses: motor signs; disability; motor complications; and cognitive status. Items are rated on a 7-point scale (from 0, normal, to 6, very severe) and total score, ranges from 0 to 24 [21], [22].

The EQ-5D is a generic, preference-based HRQoL measure [23], [24]. It includes a descriptive part of 5 items (profile), that can then be converted into an EQ-Index (from 1, perfect health state, to 0, death), and a visual analogue scale (EQ-VAS) for assessment of current health state (from 0, the worse imaginable health state, to 100, best imaginable health state).

The PDQ-8 is a specific instrument for assessment of HRQoL in PD [25]. It includes 8 items, each one scoring from 0 to 4. The PDQ-8 Summary Index is expressed as percentage of the sum of the items scores on the maximum possible scale score. Both, EQ-5D and PDQ-8 are instruments recommended for use in PD by an *ad hoc* Movement Disorder Society task force [26].

For all the aforementioned scales, with the exception of the EQ-5D index and VAS, the higher the score, the worse the assessed construct. The NMSS captures symptoms over the last month and, in patients with fluctuations, the motor and non-motor evaluations were carried out in an “on” state.

### Data analysis

Descriptive statistics were used to determine the characteristics of the sample in relation to socio-demographic aspects, historical data, and evaluations.

Using the cut-off points of the interquartile range (centiles 25, 50, and 75), the following NMSS score limits and NMSB levels were established: 0 (no NMS); 1–20 (Mild); 21–40 (Moderate); 41–70 (Severe); and ≥71 (Very severe). Once categories of the NMSS total score were established, the ability of that grouping to discriminate among patients according to the other variables in the study was determined (Kruskal-Wallis test). The difference in proportions between NMSB levels and HY stages was tested using the Goodman and Kruskal's gamma. Agreement between classifications was explored using weighted kappa with quadratic weights. Correlation between scale scores were determined by Spearman rank correlation coefficients and the difference between correlation coefficients was tested through the two tailed Fisher's z transformation. Multiple regression models were built to identify disability (SCOPA-Motor, Part B. ADL) and quality of life (EQ-5D, PDQ-8) predictors.

A post-hoc analysis was carried out using the CISI-PD as a criterion for global PD severity [21]. CISI-PD total scores have been previously categorized in global levels of PD severity (1–7 points = mild; 8–14: moderate; and ≥15 points = severe) [22] and this classification was used to create NMSS score categories to be compared with those obtained by means of centiles. Due to skewed distribution of the NMSS scores and the CISI-PD ordinal level of measure a categorical regression analysis was used to determine the association between NMSS scores and CISI-PD severity levels.

Data analysis was carried out using Stata 11.2 (StataCorp, College Station, Texas).

## Results

Nine hundred and fifty one patients, 62.6% males, were included in the study. The clinical characteristics of the sample are displayed in the Table 1. The distribution of patients by HY ratings was (n = 949): stage 1, 125 (13.2%); stage 2, 412 (43.4%); stage 3, 284 (29.9%); stage 4, 108 (11.4%); and stage 5, 20 (2.1%). Of the 835 patients with available data on treatment, 81.0% received treatment with levodopa; 58.3%, dopamine agonists; 8.0%, selegiline; 15.0%, rasagiline; 15.6%, amantadine; and 1.1%, apomorphine. Levodopa and dopamine agonists were combined in 47.3%; 44 patients (5.3%) were untreated; and 35 (3.7%) had undergone deep brain stimulation surgery for PD.

**Table 1:** Main characteristics of the sample.

	Mean	SD	Median	Range
Age at study	64.43	9.90	—	34–89
Age at onset of Parkinson's disease	56.43	10.78	—	25–89
Duration of the disease	7.99	5.78	—	0–40
SCOPA-Motor Total score	21.15	12.03	19	1–72
SCOPA-Motor A. Examination	11.64	6.61	10	0–41
SCOPA-Motor B. Activities of daily living	6.84	4.19	7	0–21
SCOPA-Motor C. Complications	2.69	3.00	2	0–12
Clinical Impression of Severity Index	8.25	4.61	8	0–24
EQ-5D index (time trade-off)	0.61	0.34	0.68	−0.65–1
EQ-5D visual analogue scale	62.43	22.11	65	0–100
PDQ-8 summary index	30.46	19.94	28.12	0–100
Non-Motor Symptoms Scale total score	50.41	41.57	39	0–225

SD: Standard deviation. SCOPA: Scales for Outcomes in Parkinson's Disease. PDQ-8: Parkinson's Disease Questionnaire-8 items.

There were 19 missing scores in NMSS domains from sixteen patients (1.7%). Due to the structure of the scale, imputation was not carried out and NMSS total score refers to the 935 patients (98.3%) with full data. The number of NMS and the NMSS scores related to each NMSB level are shown in Table 2.

**Table 2:** Non-Motor Symptoms Scale domains scores broken down by burden levels.

	Non-Motor Symptoms Burden Levels				
	No	Mild	Moderate	Severe	Very severe
	0	1	2	3	4
NMSS score range	0	1–20	21–40	41–70	≥71
**Number of NMS**	0.00±0.00	5.43±2.97	9.65±3.77	12.36±3.67	17.34±±5.15
**1. Cardiovascular**	0.00±0.00	0.36±0.84	0.79±1.70	1.46±2.31	3.98±4.76
**2. Sleep/Fatigue**	0.00±0.00	1.82±2.28	5.18±4.59	9.42±6.73	17.72±9.11
**3. Mood/Apathy**	0.00±0.00	1.06±1.63	3.97±4.56	7.34±7.33	20.52±14.26
**4. Perceptual problems**	0.00±0.00	0.20±0.90	0.52±1.41	1.10±2.73	4.50±6.00
**5. Attention/Memory**	0.00±0.00	0.81±1.26	2.73±3.58	5.59±5.98	11.70±9.65
**6. Gastrointestinal**	0.00±0.00	1.34±2.04	3.12±4.04	6.00±5.39	11.10±8.50
**7. Urinary**	0.00±0.00	1.59±2.24	4.73±4.96	8.90±8.00	16.07±10.02
**8. Sexual function**	0.00±0.00	0.49±1.53	1.79±3.25	3.74±5.11	7.44±8.30
**9. Miscellaneous**	0.00±0.00	1.34±1.84	4.86±4.88	8.34±7.60	12.99±8.81
**NMSS Total score**	0.00±0.00	9.01±4.05	27.80±6.89	51.89±7.97	106.03±34.56

* Kruskal-Wallis test for all variables, p =  0.0001.

Bonferroni correction for multiple (n = 22) comparisons, p<0.0023.

NMS: Non-Motor Symptoms. NMSS: Non-Motor Symptoms Scale.

Table 3 shows scores from other disease-related variables in the study broken down by NMSB levels. As a whole, figures indicate a significant worse state in all aspects as the NMSB level increases. The same performance was obtained with grouping by HY stages, but figures were clearly different between both methods (Table 3).

**Table 3:** Variables in the study broken down by the NMS burden levels and Hoehn and Yahr staging*.

	Non-Motor Symptoms Burden Levels				
	No	Mild	Moderate	Severe	Very severe
Level	0	1	2	3	4
**NMSS score**	0	1–20	21–40	41–70	≥71
**n** (935)	5	244	233	218	235
**PD Duration**	2.80±2.49	5.88±4.68	7.64±4.99	8.38±5.21	10.16±7.12
**SCOPA-Motor**					
A. Examination	4.00±1.87	9.54±5.16	10.35±5.56	12.16±6.11	14.89±7.94
B. ADL	0.00±0.00	4.70±3.11	5.93±3.12	7.33±3.72	9.65±4.72
C. Complications	0.40±0.89	1.43±2.27	2.28±2.55	3.07±2.80	4.11±3.57
Total score	4.40±2.07	15.68±8.85	18.55±9.04	22.56±10.68	28.57±14.35
**CISI-PD Total**	1.80±1.10	5.52±3.19	7.19±3.55	9.02±4.04	11.55±5.04
**EQ-5D Index**	1.00±0.00	0.78±0.23	0.68±0.28	0.60±0.29	0.36±0.38
**EQ-VAS**	75.80±37.43	66.73±22.65	65.08±20.86	63.11±20.86	54.35±21.62
**PDQ-8 Index**	6.25±10.60	19.88±17.85	25.80±15.89	31.51±16.87	45.70±19.05

	Hoehn and Yahr Staging				
	1	2	3	4	5
**n** (949)	125	412	284	108	20
**PD Duration**	3.77±2.55	6.91±4.68	9.24±5.38	11.75±6.84	19.10±8.93
**SCOPA-Motor**					
A. Examination	5.82±3.08	9.24±4.24	13.50±5.30	19.61±6.52	27.80±5.68
B. ADL	3.12±2.73	5.22±2.77	7.80±2.58	12.42±3.16	19.05±1.43
C. Complications	0.50±1.20	1.61±2.20	3.77±2.85	5.57±3.12	7.55±3.71
Total score	9.45±5.33	16.08±6.93	25.01±8.02	37.61±9.90	54.40±8.85
**CISI-PD Total**	3.50±2.05	6.17±2.77	10.40±3.28	14.05±3.71	18.80±3.07
**EQ-5D Index**	0.79±0.23	0.71±0.25	0.54±0.30	0.35±0.41	−0.17±0.35
**EQ-VAS**	72.22±20.33	66.70±20.32	58.10±21.50	50.30±21.92	41.25±23.00
**PDQ-8 Index**	19.52±15.85	25.16±17.02	34.79±18.90	46.03±19.20	60.94±22.25

PD: Parkinson's disease. NMS: Non-Motor Symptoms. NMSS: Non-Motor Symptoms Scale. SCOPA: Scales for Outcomes in Parkinson's Disease. CISI-PD: Clinical Impression of Severity Index for Parkinson's disease. VAS: Visual analogue scale. PDQ-8: Parkinson's Disease Questionnaire-8 items.

* Kruskal-Wallis test for all variables, p =  0.0001. Bonferroni correction for multiple (n = 18) comparisons, p<0.0027.

The correlation coefficient between motor disturbance ratings (SCOPA-Motor) and the non-motor symptoms (Non-Motor Symptoms Scale) was 0.43 (p<0.0001) in the series, ranging from 0.18 to 0.36 (weak correlation; p = 0.01–0.0001) for patients in HY stages 1 to 4 and only showing a high correlation for patients in stage 5 (r_S_ = 0.65; p<0.0001).

The corresponding distribution of patients by HY stages and NMSB levels is shown in Table 4. The agreement between these ratings of severity was weak (kappa = 0.39; CI95%: 0.37–042) and the difference in the classification of cases was statistically significant (gamma = 0.45, ASE = 0.032).

**Table 4:** Patients classification by Hoehn and Yahr staging and Non-Motor Symptoms burden levels.

Hoehn and Yahr Stages	Non-Motor burden levels					Total
	0	1	2	3	4	
1	3	55	38	19	9	124
2	2	126	122	87	67	404
3	0	55	56	81	88	280
4	0	7	16	29	54	106
5	0	0	1	2	16	19
Total	5	243	233	218	234	933

Goodman and Kruskal's gamma = 0.45; ASE = 0.032.

The following ranges and categories were obtained from the categorical regression between NMSS score and CISI-PD severity levels: 0 (absence of NMS); 1 to 15 points (mild NMS burden); 16 to 40 points (moderate NMS burden); 41 to 65 (severe NMS burden); and ≥66 points (very severe NMS burden). The coincidence between classification of patients by these levels from categorical regression and those based on the interquartile range was 858/935 (91.8%), with an agreement (kappa) = 0.97 (CI95%: 0.96–0.97). Discrepancies (8.2%) were restricted to the NMSB levels 1 (n = 55) and 3 (n = 22).

Disability, as per the SCOPA-Motor, Part B. ADL scores, was closely associated to motor impairment (motor examination + motor complications (r_S_ = 0.76), but correlated moderately with NMSS scores (r_S_ = 0.47) (Fisher's z = −11.83, 2-tailed p<0.0001). Concerning HRQoL, EQ-5D index correlated with motor impairment (r_S_ = −0.53) and NMSS (r_S_ = −0.50) (Fisher's z = 0.55; p = 0.29) and PDQ-8 scores correlated with both motor impairment and NMSS scores (r_S_ = 0.48 and 0.51, respectively; Fisher's z = 0.86; 2-tailed; p = 0.38).

After exclusion of interaction and co-linearity, age, sex, motor impairment (SCOPA-Motor, Part A. Examination), and NMSS scores were introduced as independent variables in a multiple regression model with SCOPA-Motor, Part B. ADL as dependent variable (F = 436.77; adjusted R^2^ = 0.65; p<0.0001) while similar regression models examined EQ-5D (F = 169.17; adjusted R^2^ = 0.43; p<0.0001) and PDQ-8 (F = 150.20; adjusted R^2^ = 0.39; p<0.0001) as dependent variable. The most powerful independent predictors were: for ADL, motor impairment (standardized beta = 0.68) followed by NMS (beta = 0.20); for PDQ-8 scores, NMSS scores (beta = 0.39) followed by motor impairment (beta = 0.37); and for EQ-5D motor disorder followed by NMS (motor = 0.39; NMS = 0.37). In all models, age and sex had a null or weak influence (beta<0.10).

## Discussion

The key outcomes of this study are:

1. A new strategy for clinical classification of PD patients using the NMSS in 5 stratified levels of burden (0–4 = no NMS, 4 =  very severe load of NMS, Tables 2 and 3). This simple assessment could be added to existing motor-based staging (i.e., HY) to complement PD assessment and avoid overlooking the weight of the NMS.
2. Confirmation of the significant influence of NMSB on disability and quality of life, highlighting the need to include a NMS evaluation for a complete assessment of PD patients.

This paper is aimed to propose a pragmatic, data driven clinical assessment system for PD to meet a key unmet need and a clinical challenge. We are not trying to discriminate PD patients from a control population neither is the study designed to address causation of NMS such as the role of drug therapy and pathogenesis of NMS. Instead, the NMSB classification would flag up the level of NMS load using a numerical cutoff along with motor staging to describe better the patient situation. In addition, the NMSB classification will help the physician to establish priorities in patients management taking into account disease manifestations which importance may be overlooked [16], [27].

Although NMS burden increases with increasing severity of other domains affected by PD, the correlation between motor disorder, as rated by the SCOPA-Motor, and NMS was moderate (r_S_ = 0.43), showing a loose association in all stages except the most advanced one. Most importantly, a clear discordance between motor and non-motor disturbance exists, with patients in the milder stages of motor disorder having considerable non-motor burden. For example, over one third (34.5%) of patients in HY stage 1 and 2 had severe or very severe NMSB in the present study (Table 4). This fact justifies the need, for a more complete assessment, of a specific method for classification of PD patients according to NMSB.

The proposed classification was supported by a benchmark based on clinician's global impression of severity (CISI-PD). Categorical regression allowed assignment of NMSS scores to CISI-PD ordinal categories of severity [22] and this distribution showed an excellent concordance with the proposed classification based on quartiles (agreement, 91.8%; kappa_w_ = 0.97, “almost perfect”) [28].

The importance of NMS in PD is well established and several observational and controlled studies have reported the high prevalence of NMS in PD [1], [29]–[31]. Patients, irrespective of early or advanced disease, rate NMS as one of their “first choice” symptoms of concern [32] and untreated PD patients show a high burden of NMS [30], [33]. These observations and our data confirm the fact that when questioned systematically very few patients are free of any NMS. Our cohort of patients included drug naive and advanced cases on a range of drug therapies and all HY stages (Tables 1 and 3). A wide range of patients' age was spanned in our study, although the relatively low mean age (64 years) reflects the fact that the oldest PD patients are typically not referred to the movement disorders clinics and, therefore, were under-represented in this cohort. We feel this is a reasonably representative cohort of PD patients assisted in departments of neurology and specialized units and shows how NMS occurs in early, moderately advanced and advanced PD, as also reported by the PRIAMO study [29].

We also corroborate that the burden of NMS is a key determinant of quality of life in PD [2], [29], [34], [35] and indeed this is one of the key outcomes of this work. This is illustrated by the regression models where the burden of NMS appears to be as important a predictor of quality of life (EQ-5D and PDQ-8) as is motor impairment. Interestingly, when both motor and non-motor impairment are taken into account, age had a negligible influence on quality of life, a finding that emphasises the considerable impact of both motor and non-motor disabilities on patients. These are important issues as the basis of assessment and therapeutic strategies in PD should be driven by impact on quality of life [36], [37].

What then could be the clinical implications if NMSB classification as proposed in this paper is adopted in “real life” practice? Firstly the proposed numerical grading should help improved patient care by alerting the clinician for the need for addressing treatable NMS [1]. In current clinical practice, NMS burden is often not assessed and a European study reported that various NMS were never declared to health care professionals in 60% of cases compromising care [16]. Secondly, the statistical concordance between HY staging and NMS classification denotes only weak agreement (when *k* = 0.21 to 0.40) [28] between these two assessment paradigms. For instance, as shown in Table 4, only 5 patients in HY stages 1 or 2 reported no NMSB whereas 76 experienced NMSB grade 4 (very severe). Conventionally, HY stage 1 and 2 represent mild PD, but this qualification cannot be supported attending the load of NMS, any domain/s they belong. The non-motor manifestations present in PD may be very variable in number and type and they maintain only a moderate association with the motor disturbances. Clinical and neuropathological data are now emerging supporting our concept of the aforementioned non-motor dominant endophenotype and the clinical heterogeneity of PD [5], [33], [38].

The strength of this analysis is the large number of patients included spanning early and untreated to very advanced motor disease and a range of therapies. However, there are obvious limitations to this exploratory work. Firstly we do not have control data for this study although in the validation of our NMS instruments a collection of healthy control data was obtained showing that while some NMS such as insomnia, nocturia and pain may be common amongst controls, the severity of these symptoms is considerably greater in PD [39]. Additionally, the proposed classification is not aimed at discrimination of PD from controls and it would not be feasible to apply the HY classification to a control population.

An important limitation of the study is its selection bias, as patients were recruited from specialized units of tertiary centres and, therefore, findings may not be generalizable to the entire PD population. The most advanced phases of disease were under-represented and this may have influenced results leading, for example, to false high correlations between motor and non-motor problems for HY stage 5.

Like many studies evaluating symptoms, we used a relatively “cognitively intact” cohort. As such one may argue that lack of inclusion of patients with dementia would be a drawback of this study. We would, however, point out that only patients with “overt” dementia were excluded and the sample studied includes patients with cognitive impairment and would be reflective of PD population normally seen in clinics spanning untreated to advanced PD. This approach was required as the NMSS and other scales would be difficult to complete in patients with frank dementia.

NMSS relates to symptoms over the “last month”. The assessments are done largely in “on” state and reflect the fact that this work is aimed at a “global” assessment which encapsulates “on” and “off” phases while recording NMS. Furthermore, also were included patients in early disease states who are either untreated or do not have clearly defined on and off fluctuations. Nonetheless, in the present study, comparison between the NMS levels and the motor HY staging (Table 4) is only valid for patients "on" medication, as the "off" medication state was not considered and this fact could substantially modify the results.

It is possible that NMS recorded may be modified by dopaminergic therapies however, in this study as we are evaluating motor and NMS at the point of examination, it is irrelevant if the patient has drug related NMS as this study cannot identify the cause of the NMS. Additionally, the identification of a high NMS load in such a case, for instance, may allow the clinician to address drug related causation.

We, therefore, propose that a combined motor staging (with HY) and NMS burden classification (from grade 0–4) be adopted for use in clinical practice. Further large scale longitudinal and clinico-pathological correlation studies are now required to define the prognostic and clinical value of the NMSB grading strategy.

## Supporting Information

Annex S1 **Contributors to the studies from which data used in the present study were generated.**
(DOC)
