# Correction: A Proposal for a Comprehensive Grading of Parkinson's Disease Severity Combining Motor and Non-Motor Assessments: Meeting an Unmet Need

**DOI:** 10.1371/journal.pone.0150130

**Published:** 2016-02-19

**Authors:** Kallol Ray Chaudhuri, Jose Manuel Rojo, Anthony H. V. Schapira, David J. Brooks, Fabrizio Stocchi, Per Odin, Angelo Antonini, Richard G. Brown, Pablo Martinez-Martin

The eighth author’s name is misspelled. The correct name is: Richard G. Brown.
