# Supplementary material for: A Proposal for a Comprehensive Grading of Parkinson's Disease Severity Combining Motor and Non-Motor Assessments: Meeting an Unmet Need
Source: PLoS One. 2013 Feb 27;8(2):e57221. doi: 10.1371/journal.pone.0057221 (PMC3584126; doi:10.1371/journal.pone.0057221)
Supplement: Annex S1 — Contributors to the studies from which data used in the present study were generated. (DOC) [file pone.0057221.s001.doc]

**Annex S1**

**Contributors to the studies from which data used in the present study were generated**

| **Country** | **Principal Investigator** | **Center** |
| --- | --- | --- |
| Italy | F Stocchi | IRCCS San Raffaele, Rome, Italy |
| Kansas, USA | K Lyons | University of Kansas Med. Ctr., Kansas City, KS, USA |
| Spain | M Kurtis | Hospital Ruber Internacional, Madrid, Spain |
| Germany | P Odin | Bremerhaven Hospital, Germany |
| Ecuador | M Serrano | Hospital Carlos Andrade Marin, Quito, Ecuador |
| Brasil | V Borges | Universidade Federal de Sao Paulo, Sao Paulo, Brazil |
| India | M Behari | All India Institute of Medical Sciences, New Delhi, India |
| India | K K Bhattacharya | RG Kar Medical College, Kolkata |
| Greece | S Bostantjopoulou | Arsitotle University of Thessaloniki, Thessaloniki, Greece |
| Israel | JM Rabey | Asaf Harofeh Medical Center, Zerifin, Israel |
| Argentina | F Micheli | José de San Martín Clinical Hospital, Buenos Aires, Argentina |
| Mexico | M Rodriguez Violante | Instituto Nacional de Neurologia y Neurocirugia, Mexico DF, MX |
| Cuba | M Alvarez | CIREN, La Habana, Cuba |
| UK | G Macphee | Southern General Hospital, Glasgow, UK |
| UK | B Kessel | South London NHS Trust, UK |
| UK | P Worth | Norfolk and Norwich University Hospital, UK |
| UK | C Ellis | Medway University Hospital, UK |
| UK | F Martin/T Andrews | St Thomas’ Hospital, London |
| UK | M Silverdale | Manchester University Hospital, UK |
| Austria | R Katzenschlager | Danube Hospital/SMZ-Ost, Vienna, Austria |
| Portugal | M Coelho | Hospital Santa Maria, Lisbon, Portugal |
| Italy | G Riboldazzi | Center for Parkinson’s Disease, Macchi Foundation, Varese, Italy |
